# Honeybees (*Apis mellifera*) Learn Color Discriminations via Differential Conditioning Independent of Long Wavelength (Green) Photoreceptor Modulation

**DOI:** 10.1371/journal.pone.0048577

**Published:** 2012-11-14

**Authors:** David H. Reser, Randika Wijesekara Witharanage, Marcello G. P. Rosa, Adrian G. Dyer

**Affiliations:** 1 Physiology Department, Monash University, Clayton, Victoria, Australia; 2 School of Media and Communication, Royal Melbourne Institute of Technology, Melbourne, Victoria, Australia; University of Sheffield, United Kingdom

## Abstract

**Background:**

Recent studies on colour discrimination suggest that experience is an important factor in how a visual system processes spectral signals. In insects it has been shown that differential conditioning is important for processing fine colour discriminations. However, the visual system of many insects, including the honeybee, has a complex set of neural pathways, in which input from the long wavelength sensitive (‘green’) photoreceptor may be processed either as an independent achromatic signal or as part of a trichromatic opponent-colour system. Thus, a potential confound of colour learning in insects is the possibility that modulation of the ‘green’ photoreceptor could underlie observations.

**Methodology/Principal Findings:**

We tested honeybee vision using light emitting diodes centered on 414 and 424 nm wavelengths, which limit activation to the short-wavelength-sensitive (‘UV’) and medium-wavelength-sensitive (‘blue’) photoreceptors. The absolute irradiance spectra of stimuli was measured and modelled at both receptor and colour processing levels, and stimuli were then presented to the bees in a Y-maze at a large visual angle (26°), to ensure chromatic processing. Sixteen bees were trained over 50 trials, using either appetitive differential conditioning (N = 8), or aversive-appetitive differential conditioning (N = 8). In both cases the bees slowly learned to discriminate between the target and distractor with significantly better accuracy than would be expected by chance. Control experiments confirmed that changing stimulus intensity in transfers tests does not significantly affect bee performance, and it was possible to replicate previous findings that bees do not learn similar colour stimuli with absolute conditioning.

**Conclusion:**

Our data indicate that honeybee colour vision can be tuned to relatively small spectral differences, independent of ‘green’ photoreceptor contrast and brightness cues. We thus show that colour vision is at least partly experience dependent, and behavioural plasticity plays an important role in how bees exploit colour information.

## Introduction

Colour vision allows pollinating insects to detect and discriminate flowers on the basis of reflected wavelengths, independent of potential brightness differences [1] such as those occurring in complex natural environments [2]. Indeed, there is strong evidence that flowering plants (angiosperms) have evolved spectral signals that are optimally discriminated by the visual system of hymenopterans capable of trichromatic vision [3]; [4]. The classic model for understanding colour vision in hymenopteran insects is the honeybee [5]–[14]. Individual bees tend to be flower constant, and will visit one type of flower as long as it is easily ‘discriminated’, facilitating efficient delivery of species-specific pollen and thus driving flower evolution [15].

Honeybee trichromatic vision is based on an ‘ultraviolet’ (UV) photoreceptor, which is maximally activated by 344 nm radiation, a ‘blue’ photoreceptor (maximally activated by 436 nm radiation), and a ‘green’ photoreceptor (maximally activated by 544 nm radiation) [16]. To characterise a colour vision system in a given organism, it is necessary to empirically determine the Δλ/λ function, which describes the differences in wavelength that can be resolved in various regions of the electromagnetic spectrum [17]. The Δλ/λ function in free flying honeybees [18] yields the finest chromatic discrimination at wavelengths near 390 and 490 nm. Conversely, in the band of wavelengths between 425 and 475 nm, discrimination is relatively poor. This behavioural result dovetails with calculations based on measurements of peak absorbance and bandwidth in the three colour photoreceptor types in this species [3]; [16].

An important question regarding colour vision is whether it is entirely mediated by “hard-wired” physiological mechanisms [19], or is partially learned via individual experience [20]. In honeybees, two recent studies have shown that colour vision is modifiable with experience for perceptually similar colour stimuli. In the first study, free flying honeybees were trained on a colour discrimination task in a Y-maze apparatus using either absolute or differential conditioning [21]. Absolute conditioning involves training on a target presented in isolation, while differential conditioning presents the target stimulus in relation to a perceptually similar stimulus. Bees trained using absolute conditioning could reliably discriminate very dissimilar stimulus colours, but generalised similarly coloured stimuli [21]. In contrast, bees provided with differential conditioning could learn much finer discriminations, albeit slowly [21]. A second study tested whether the association of an aversive tasting substance with a distractor stimulus influenced learning, and concluded that honeybees can learn to resolve very similar colours if a combination of appetitive and aversive differential conditioning was used [22]. These studies suggest that colour discriminations are shaped differently depending upon individual experience [21]–[23]. However, as detailed below, a potential confound in both studies was the use of stimuli with the potential for incongruent modulation of the ‘green’ photoreceptor during differential conditioning.

Interpretation of behavioural experiments on colour vision with honeybees can be difficult due to the complexity of the colour visual pathway [20], with different colour channels employed in different contexts depending upon the subtended visual angle of the stimulus [24]; [25], or upon motion cues [26]. While the ‘UV’, ‘blue’ and ‘green’ photoreceptors all contribute to chromatic perception via opponent processing [9]; [20], these channels can invoke varying patterns of activation in the brain. Thus, the “UV” and “blue” photoreceptor outputs project directly to the second optic ganglia of the bee brain (the outer medulla) [27]; [28], while the ‘green’ photoreceptor signal is initially processed in the first optic ganglia (the lamina) [29]–[31]. Furthermore, behavioural experiments have shown that modulation of the ‘green’ photoreceptor is responsible for stimulus detection at small visual angles [24]; [32]; [33], broad field motion processing (as measured with the optomotor response) [34], small field motion detection at high frequencies [35]; [36], and some complex pattern recognition tasks, which can be learned only through differential conditioning [37].

It is currently not clear if previously described learning of fine color discriminations [21]; [22] can be explained solely on the basis of a simple mechanism based on the presence of contrast in the ‘green’ photoreceptor channel, or actually depends on differential conditioning and more complex colour processing that suggests neural plasticity depending upon individual experience, or tuning of attention. To evaluate these possibilities, we assessed the ability of free-flying honeybees to learn to discriminate perceptually similar chromatic stimuli which exclude modulation of the long wavelength ‘green’ photoreceptor, and were presented at a visual angle that predominantly stimulates the chromatic processing system of the honeybee. This work provides important insights into the behavioural flexibility of bees and other insects, and provides indirect information about possible neural structures involved in colour learning [20]; [38].

## Materials and Methods

**Figure 1:**
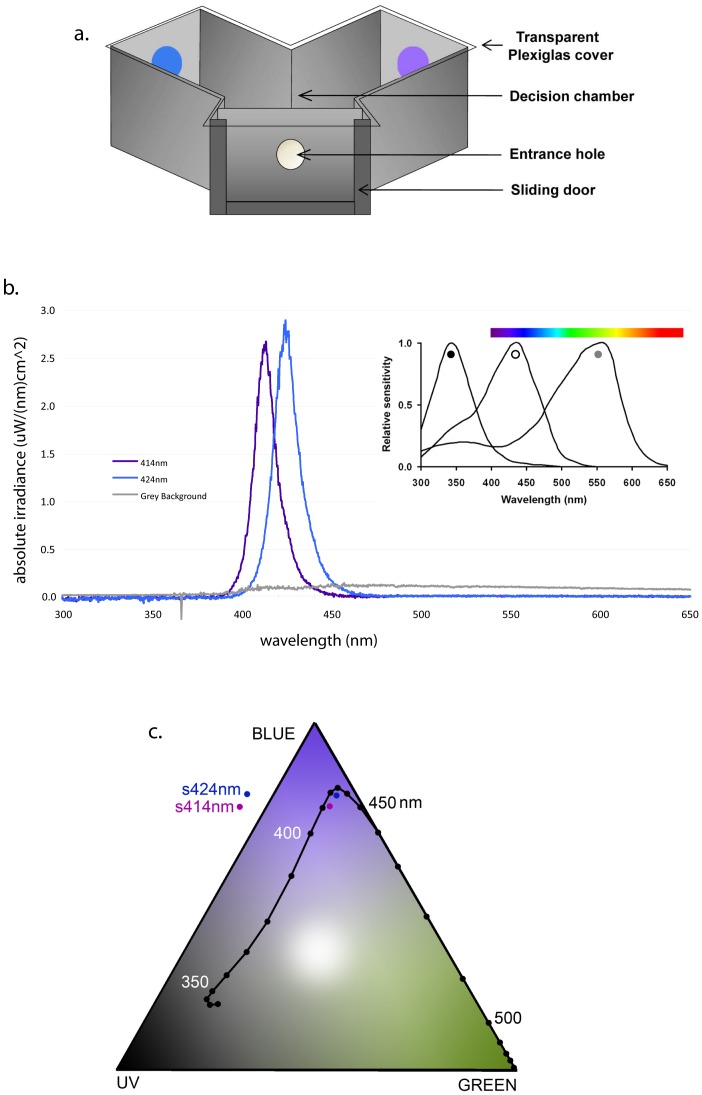
Testing of honeybee colour vision. (a) Schematic diagram of Y-maze apparatus used to test free flying bees. LED array position was varied between experimental trials (see Methods). (b) Example of an absolute irradiance spectra measurement for LED stimulus arrays. Inset shows spectral sensitivity for long- (grey circle), medium- (open circle), and short- (black circle) wavelength sensitive photoreceptors in honeybees. Colour bar indicates approximate perceived colour range in humans. Adapted from Dyer et al., 2011 using the data for honeybees from Peitsch et al. 1992. (c) Plots of LED stimuli in a Maxwell triangle colour model representing the trichromatic (ultraviolet, UV; blue, B; green, G) colour vision of honeybees. Spectral loci show theoretical stimulation by spectrally pure radiation.

Experiments were performed outdoors in the Jock Marshall Reserve at Monash University. Free-flying honeybees (*Apis mellifera*) were allowed to visit a gravity feeder containing 10% (vol/vol.) sucrose solution [39]. Experiments were conducted during summer on days of low wind [40] with ambient temperature between 20–30°C. The colour temperature of the foraging environment was approximately 6500°K [41]; [42]. Individual bees were recruited from the gravity feeder to a nearby testing site, marked on the dorsal abdomen for identification, and trained to enter a Y-maze apparatus (Figure 1) to collect a 30% sucrose reward.

### Stimuli

Target and distractor stimuli each consisted of constantly illuminated arrays of 76 light emitting diode (LED) bulbs [individual bulb diameter 5 mm, 410 nm (405–415 nm half-bandwidth) or 420 nm (415–425 nm) manufacturer specified peak wavelength; VioLED, Inc., Taichung, Taiwan, ROC]. The LEDs were controlled on independent channels using a purpose built electronic control box. Each LED array was placed behind a 3 mm thick sandblasted UV-transparent plexiglas diffuser, yielding an evenly illuminated circular target of 7 cm diameter. During experiments each stimulus was positioned in a Y-maze (Figure 1a) arm at 15 cm from the decision chamber. This viewing distance equates to a visual angle of 26° at the decision line in the Y-maze. In addition, these stimuli encompassed an angle greater than 15° from any position in the decision chamber, thus always exceeding the visual angle required to stimulate the chromatic pathways in the honeybee visual system [10]; [24]; [25]; [43].

The peak wavelength separation between the LED stimuli was selected to ensure that the colour discrimination task would by perceptually difficult, being close to the known limit of discrimination ability for the honeybee [18]. The output of the LED stimuli and diffuser combination was sampled by absolute irradiance measurement using a spectrophotometer (model S2000, Ocean Optics, Dunedin, FL, USA) calibrated against known deuterium and halogen light sources (DH-2000-CAL, Ocean Optics, Dunedin, FL, USA) [44]; [45]. The background material surrounding the LED stimuli consisted of a grey card (HKS 92N) which was also measured with the spectrophotometer in the experimental conditions specified above.

Absolute irradiance spectra had peak wavelengths for respective stimuli of 413.9 and 424.4 nm for the ‘410’ and ‘420’ nm manufacturer-specified bulbs (Fig. 1b); LED stimuli are thus described as 414 and 424 nm for the remainder of this report. Absolute irradiance spectra were obtained at a range of outputs using the control box, and the input-output function for each LED array was then modelled for relative stimulation of the honeybee colour visual system by converting irradiance spectra to relative quantum flux, and numerically integrating at 10 nm steps with published data for honeybee photoreceptor sensitivity [16]. Power settings were calibrated so that there was less than a 3% variation in the summed brightness to the ‘UV’, ‘Blue’ and ‘Green’ photoreceptors for the chromatic processing system (equations 1–3), whilst modulation of the ‘Green’ photoreceptor was less than 5% (equation 4). This was achievable, as stimuli were specifically chosen to minimally stimulate the beta peak of the ‘green’ photoreceptor; lying in the ‘flat’ region of the trough between the alpha- and beta-peaks [46]; there was in fact little overall activation of the ‘green’ photoreceptor compared to the shorter wavelength photoreceptors (Fig. 1b-inset). Previous behavioural experiments suggest that the colour visual system of honeybees does not process brightness as a dimension in chromatic perception [5]; [18]; [47]–[50]; thus, the 3% variation in total stimulus intensity we empirically measured is negligible. In addition, analysis of behavioural experiments for honeybees vision suggest that brightness differences need to be greater that 14–20% for a detection task even at the correct visual angle (<15°) for the ‘green’ photoreceptor modulation [24]; [51], above 7.2% in a optomotor movement task [34], or ‘high’ for shape recognition tasks [52]. Indeed, studies on the potential use of Green-receptor contrast by honeybees at a large visual angle show that input is negligible due to low sensitivity [25]; [53], especially when strong chromatic cues are also present [53]. In agreement with these findings, experiments on another trichromatic insect, the hawkmoth (*Macroglossum stellatarum*), shows that insect colour vision does not separately evaluate ‘green’ contrast cues in the presence of chromatic cues even when long-wavelength ‘green’ photoreceptor modulation is changed by a factor of 10 [54]. In summary, under our experimental conditions with stimuli that strongly modulate differences in the honeybee UV- and Blue- photoreceptors, but do not modulate the Green-receptor to above known threshold values, only the chromatic processing pathways could potentially enable learning of differences in stimulus properties.
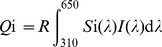
(1)


Where *Q* is the quantum catch for ‘i’ = (1:UV, 2:Blue, 3:Green) photoreceptors respectively, *S*i(λ) is the spectral sensitivity function of a particular photoreceptor according to data for honeybees [16], and *I*(λ) is the relative spectral photon flux of stimuli. The adaptation state of the visual system follows a von Kries normalisation (R; equation 2) [42].
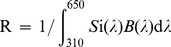
(2)where *B*(λ) is the is the relative spectral photon flux of the background and ‘i’ = 1–3.
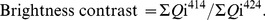
(3)where ‘i’ = 1–3.
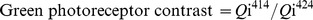
(4)where ‘i’ = 3.
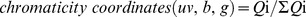
(5)where ‘i’ = 1–3 and thus *uv*+*b*+*g* = 1.0 (Table 1).

**Table 1:** Colour distance in a Maxwell colour triangle for ‘colour’ stimuli that are only learnt by free flying honeybees that experience differential conditioning.

Study	Stimulus pair	Triangle colour distance
Giurfa 2004 [20]	HKS 37N vs 43N	0.089
Avarguès-Weber et. al 2010 [21]	HKS 43N vs 47N	0.071
Avarguès-Weber et. al 2010 [21]	HKS3N vs 68N	0.084
Dyer & Murphy 2009 [22]	Similar ‘blues’	0.043
Current	414 vs 424 nm	0.039

The stimuli used in the current study are more perceptually similar than previous studies.

The chromaticity coordinates (equation 5) were used to plot the loci of the respective test stimuli in a Maxwell Colour Triangle as this colour model is independent of brightness effects between stimulus and the background [11]; [41]; [55]–[58]. This model shows that the stimuli have a negligible influence on the ‘Green’ photoreceptor (Fig. 1c) which is because the stimuli lie in the saddle of the beta-peak of the ‘Green’ photoreceptor (Fig. 1b). To enable a comparison of the current data to three previous studies that have already shown that differential conditioning is important to colour learning in honeybees (but did not specifically exclude ‘Green’ contrast), we calculated the Euclidean colour distance in the Maxwell colour triangle (Table 2).

**Table 2:** Chromaticity coordinates (*uv*, *b*, *g*) which are the normalised photoreceptor quantum catches [P(UV, Blue, Green)] for a given stimulus such that *uv*+*b*+*g* = 1.0.

Stimulus	*uv*	*b*	*g*
**414 nm**	0.136	0.752	0.112
**424 nm**	0.079	0.801	0.120

In order to ensure that the use of LED stimuli did not introduce any new effects on learning relative to previous behavioural studies in which reflective (colour card) stimuli were employed, we replicated behavioural experiments which have previously been described for card stimuli and have shown bees are relatively insensitive to brightness differences for stimuli presented at a large visual angle [21]; [53]. In the first of these control experiments, bees were given appetitive absolute conditioning for 20 trials to either the 414 nm or 424 nm stimuli (4+4 bees counterbalanced), and then transfer tests that presented (i) both 414 nm and 424 nm stimuli, and (ii) the target stimulus (414 nm or 424 nm) versus a novel stimulus comprised of white LED filtered by a transparent HKS 3N yellow filter. In the second control experiment, bees were provided with appetitive differential conditioning to the 414 nm and 424 nm stimuli (counterbalanced) for 50 trials and then provided with (i) a learning test, (ii) a transfer test that presented the target stimulus at a 50% increase in stimulus brightness, and (iii) a transfer test that presented the distractor stimulus at a 50% increase in stimulus brightness.

### Behavioral testing

Reward or neutral/aversive solutions were placed on small platforms 1 cm in front of the respective LED stimuli. The platforms were made of thin wire and were identical, so did not provide a visual cue. Previous work has also shown that these solutions do not provide olfactory cues for bees [22]. The 414 and 424 nm stimuli were alternated as the target and distractor between bees in a counterbalanced experiment design. Each bee was trained for 50 trials, which is a training length that has previously been reported for fine colour and spatial discrimination tasks with differential conditioning in bees [20]; [44]; [59]–[62]. The first choice, defined as crossing the decision line in the Y-maze, of the bee in each trial was recorded and used for statistical analysis [63]. Correct identification of the target LED stimulus was rewarded on each learning trial with a 30% (vol./vol.) sucrose solution reward, while incorrect choices yielded either a neutral (tap water) or aversive (aqueous 60 mM quinine HCl) stimulus. After each trial, the contact surfaces and Y-maze were cleaned with 10% ethanol to preclude the use of scent marking cues on subsequent trials. Stimuli were presented in alternative Y-maze arms in a pseudo random order. At the conclusion of the 50 trial training cycle, each bee received 45 s duration unrewarded touch tests counterbalanced within subject for each arm of the Y-maze, during which the number of touches on the empty reward platform were recorded. This touch test allows for the collection of non rewarded data to exclude all possible confounds like scent marking. Between the respective touch tests, a refresher trial was presented to ensure that the bee remained motivated for the subsequent test as has been previously described [22]; [63].

### Data analysis

Performance during the 50 trial training cycle was evaluated by calculating the frequency of correct choices across 10 blocks of 5 trials, and the accuracy of the learned color discrimination was assessed from performance on the unrewarded touch tests within each group (neutral and aversive). Acquisition curves were plotted for each reward condition, and these data were compared to the trial block data using a 2-way repeated measures ANOVA procedure. Unrewarded touch test data were evaluated using the Wilcoxon signed rank test against an expected median correct choice frequency of 50% (chance level), and the different treatments (quinine vs. water) were compared using the Mann-Whitney U test.

## Results

**Figure 2:**
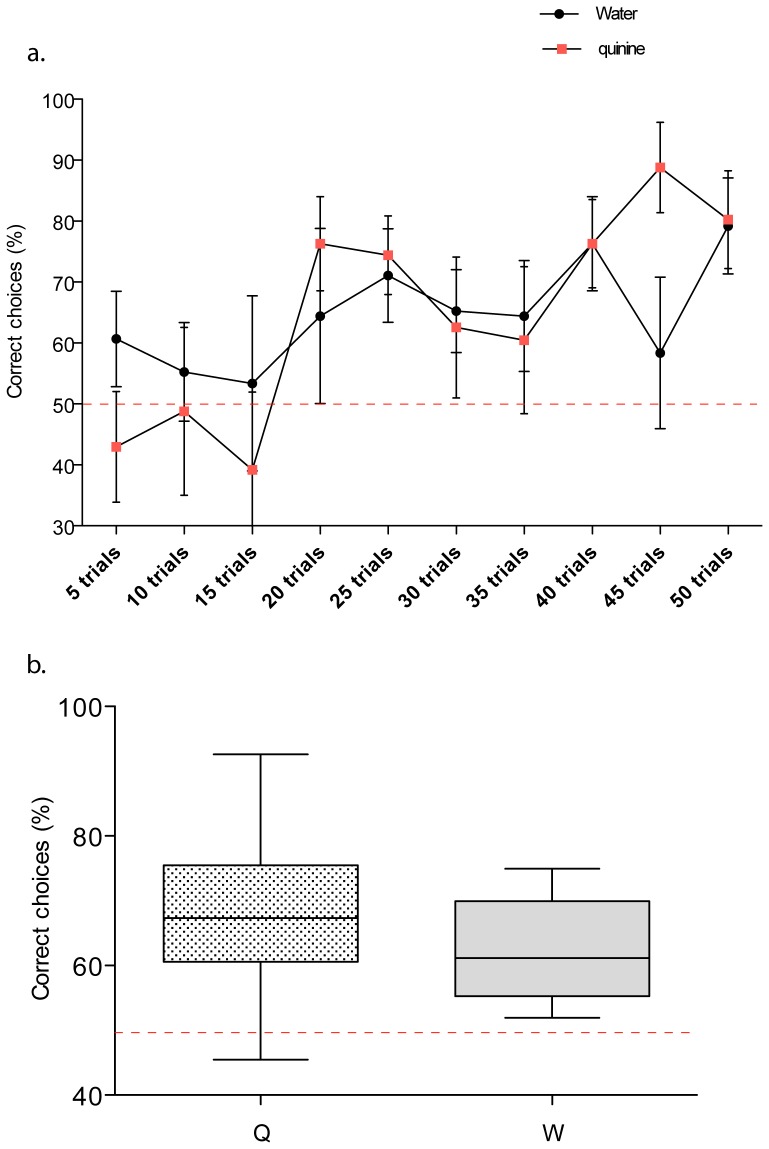
Colour learning in honeybees with differential conditioning. (a) Acquisition curves for LED colour discrimination over 50 trials. Mean ± SEM of correct choice frequency for each 5-trial block. (b). Unrewarded touch test performance following 50 trial training interval for neutral (W: water) and aversive (Q: quinine) distractor stimuli. Mean ± SD correct touch frequency. Note that the Y-axis in each panel is abbreviated to enhance visibility. Red dotted lines indicate expected chance performance level (50%) bee choices following differential conditioning were significantly different from chance (see text for stats). In the touch tests the mean number of choices made by bees was 29.4±1.4 SEM.

Acquisition performance for the color discrimination task over the 50 trial training cycles are presented in Figure 2a. Both the neutral (water) and aversive (quinine) distractor stimuli resulted in approximately 80% frequency of correct choices in the final trial block (trials 45–50; mean frequency of correct choice water = 79%, quinine = 80% diff = 1%, 95% CI −39–40%, ns). Improvement of correct choice frequency with repeated trials was significant for both treatment groups (2-way ANOVA, trial block X treatment, df = 9,1, trial block F = 2.9, p = 0.006). The learning curves clearly show that differential conditioning was important to the task as an increase in acquisition only begins after 15 trails, whilst previous empirical studies report that with absolute conditioning high levels of colour learning occur in fewer that 8 trials [21]; [50]; [64]. This behavioural finding is supported by the colorimetric modelling which shows that the colour distance between the 414 and 424 nm test stimuli was less than the colour distances between stimuli for which previous work has shown differential conditioning is important for colour learning in free flying honeybees (Table 2).

The overall rate of acquisition and correct choice frequency after the final trial block were similar for the aversive and neutral stimuli (2 way ANOVA, trial block X treatment, df = 9,1, treatment F = 0.001, p = 0.977), suggesting that learning of this colour discrimination task with differential conditioning was independent of aversive reinforcement. This conclusion is also supported by the relative performance of the neutral and aversive stimulus groups in the unrewarded touch test procedure. Figure 2b summarises the outcome of the post-training assessment of learning accuracy, and for both the quinine (z = −2.38, d.f. = 7, p = 0.018) and water groups (z = −2.52, d.f. = 7, p = 0.012) correct choice frequency was significantly different from the expected 50% level by the Wilcoxon signed rank test. No difference was observed in frequency of correct touches between the neutral and aversive distractor conditions (median and 95% confidence quinine: 68.4%, 52.8–77.1%, water: 64.4%, 58.7–70.9%; Mann-Whitney U = 20.5, 2 tailed p = 0.248, ns). Data and LED spectra are logged at Dryad; DOI: doi:10.5061/dryad.g2r32

### LED control experiments

As the current study required the use of LEDs as stimuli in order to both produce narrow-band spectra, and at the same time be displayed at a large visual angle, the following tests were conducted to test that this methodology elicited colour learning consistent with previous experiments employing coloured card stimuli.

In the first LED control test, N = 8 honeybees were given appetitive absolute conditioning for 20 trials to either the 414 nm or 424 nm stimuli (4+4 bees counterbalanced), followed by transfer tests comparing the target to (i) the similar distractor, and (ii) to a previously unseen yellow LED, as described in the Methods section. This experiment established that in transfer test (i) bees could not discriminate between the similar target and distractor stimuli (414 nm and 424 nm) at a level significant from chance expectation (56.3%+/−3.6 SEM; 1-sample t-test, t = 1.739, d.f. = 7, p = 0.126), but (ii) these bees had learnt to reliably discriminate the target from a novel yellow stimulus (95.8%+/−1.2 SEM, t = 14.547, d.f. = 7, p<0.001). Two of these bees were also given a further 40 trials learning opportunity with the target stimulus; neither bee scored greater than 55% accuracy on a final transfer test presenting the 414 nm or 424 nm stimuli.

In the second LED control test, a separate group of N = 8 bees were provided with appetitive differential conditioning to the 414 nm and 424 nm stimuli (counterbalanced) for 50 trials, followed by a learning test and two transfer tests which respectively varied the target/distractor stimulus brightness by 50%, as described in the Methods. In all three tests the target stimulus was preferred, and there was no significant difference between the three respective tests [test (i; learning)** = **69.7%+/−3.3 SEM; test (ii; target increased brightness) = 71.8%+/−3.1 SEM; test (iii; distractor increased brightness) = 73.4%+/−3.4 SEM; one way repeated measures ANOVA Wilks' lamda = 0.879, F(2,6) = 0.414, p = 0.679, partial eta squared = 0.121] showing that honeybees used chromatic information rather than brightness as a cue to make the discrimination between these stimuli when presented at a large visual angle.

These two control experiments are consistent with previous studies that have used coloured cards to evaluate colour processing in free flying honeybees [21]; [53], indicating that the use of LED based stimuli in this experiment did not introduce any additional learning artefacts for colour experiments with free flying honeybees.

## Discussion

A number of recent studies suggest that the way in which insects, including: honeybees [21]; [22]; [65], bumblebees [44]; [59]; [60], hawkmoths [66], and ants [67] learn colour discriminations is dependent upon the type of visual conditioning procedure employed. Colour vision is defined as the capacity of a visual system to discriminate between colours based on spectral differences, independent of intensity [1]; [68]. The current study shows that in honeybees, the capacity to learn spectral differences following extended differential conditioning is independent of modulation the ‘green’ photoreceptor and overall stimulus brightness. This demonstrates for the first time that differential conditioning directly affects learning for colour vision. This finding is relevant for understanding how honeybees, and probably other insect pollinators, find ‘colourful’ flowers in complex natural environments, where there are likely to be large and frequent variations in brightness cues available to achromatic visual systems. Indeed, the current result is consistent with recent findings in bumblebees that differential conditioning can yield very fine spectral differences for stimuli that do not specifically modulate the ‘green’ photoreceptor channel, i.e. flower petal iridescence [62]. The plastic colour visual system of honeybees is likely to confer an advantage for making fast decisions in foraging situations where there are salient differences between rewarding and non rewarding flowers, as well as potentially reducing energy loss in scenarios where mimic flowers might closely replicate the colour of a rewarding model flower [4]; [20]; [69].

An interesting observation in this study was the absence of a significant difference in performance between groups trained with either appetitive differential conditioning, or aversive-appetitive differential conditioning (Fig. 2). This finding indicates that even though the colour task provided to the bees was perceptually difficult and required differential condition to learn, the task difficulty was unlikely to be at the limit of honeybee colour discrimination. This explanation is consistent with a previous report that honeybees trained with extensive aversive and appetitive differential conditioning can learn to discriminate a colour distance of 0.008 hexagon units [12]. We thus do not conclude that the association of an aversive stimulus with the distractor stimulus in differential conditioning paradigms is ineffective, but rather that the discrimination task in this current experiment was sufficiently easy as to not warrant aversive stimulation [22].

The current study shows that colour vision in bees is not exclusively mediated by mechanistic or hard-wired elements, rather it is a plastic behavioural response, modifiable with specific visual experience. Interestingly, colour vision in vertebrate visual systems has also recently been shown to have a large degree of plasticity. Dichromatic mice genetically altered to express a human red photopigment instead of the mouse green pigment on the X-chromosome exhibit perception of an extended spectral range, when provided with extensive differential conditioning [70]. Further evidence of vertebrate behavioural plasticity for colour processing was provided by gene therapy experiments which added a third cone photopigment to dichromatic squirrel monkeys, which resulted in acquired trichromatic visual behaviour even in mature monkeys [71]. This level of behavioural plasticity for colour learning in both vertebrates and invertebrates is surprising, as it requires considerable neural plasticity within a very short time frame for the individual to learn to use the spectrally different signals. However, the evidence that mice and monkeys can tune colour vision following differential conditioning in a couple of days [70]; [71], and honeybees can learn new colour discrimination abilities in a matter of hours, suggests that colour vision plasticity is associated with a strong evolutionary advantage.

A critical issue which remains to be addressed is where in the brain learning of colour information may occur. The current result suggests that the locus or loci is not at an early stage of visual processing, since it is known that the UV and Blue photoreceptors first project to the 2^nd^ optic ganglia (medulla) in the honeybee [20]; [27]. Electrophysiological and neuroanatomical studies on colour processing in bumblebees [28]; [72]; [73] suggest that there may be two separate colour pathways in the bee brain: a relatively hard wired neural projection from the outer layers of the medulla and lobula to the posterior protocerebrum which allows for coarse colour discriminations, and a modifiable second pathway via the anterior lateral protocerebrum which could potentially allow for colour learning [20]. Intracellular recordings from neurons along the posterior pathway display colour sensitive and colour opponent response, but show little adaptation to temporal variations from LED stimuli [72]; [73]. In contrast, neurons along the lateral protocerebrum pathway exhibit colour sensitivity, colour opponency and temporally complex patterns including adaptation and entrainment [28]; [72]–[74]. This leads to the testable hypothesis that elements of the lateral protocerebrum pathway could include neural correlates of the behavioural plasticity observed following either absolute or differential conditioning to colour stimuli [20]. Recent studies of neuroanatomical and neurophysiological correlates of colour information processing in the anterior optic tubercle, a region within the lateral protocerebrum of the honeybee brain, suggest that this region contains different subunits that reflect segregation of complex colour information [38]. Understanding how these neural circuits respond and adapt to different types of chromatic information depending upon individual experience could yield insights into the neural bases that of behavioural plasticity observed in free flying bees, and this represents a fertile target for future investigations.

## References

[1] Chittka L, Wells H (2004) Color vision in bees: mechanisms, ecology and evolution. In: Prete F, editor. How simple nervous systems create complex perceptual worlds. Boston: MIT Press. pp. 165–191.

[2] Chiao C-C, Cronin TW, Osorio D (2000) Color signals in natural scenes: characteristics of reflectance spectra and effects of natural illuminants. J Opt Soc Am A 17: 218–224. 10.1364/josaa.17.000218 10680623

[3] Chittka L, Menzel R (1992) The evolutionary adaptation of flower colors and the insect pollinators' color vision systems. J Comp Physiol A 171: 171–181.

[4] Dyer AG, Boyd-Gerny S, McLoughlin S, Rosa MGP, Simonov V, et al (2012) Parallel evolution of angiosperm colour signals: common evolutionary pressures linked to hymenopteran vision. Proc Royal Soc B In press. doi:10.1098/rspb.2012.0827. 10.1098/rspb.2012.0827 PMC3396912 22673351

[5] Daumer K (1956) Reizmetrische Untersuchung des Farbensehens der Bienen. Z Vergl Physiol 38: 413–478.

[6] von Frisch K (1914) Der Farbensinn und Formensinn der Biene. Zool Jb (Physiol) 37: 1–238.

[7] Spaethe J, Tautz J, Chittka L (2006) Do honeybees detect colour targets using serial or parallel visual search? Journal of Experimental Biology 209: 987–993. 16513924 10.1242/jeb.02124

[8] Srinivasan M, Lehrer M (1985) Temporal resolution of colour vision in the honeybee. J Comp Physiol A 157: 579–586. 3837099 10.1007/BF01351352

[9] Menzel R, Backhaus W (1991) Colour vision in insects. In: Gouras P, editor. The Perception of Colour. London: Macmillan Press. pp. 262–293.

[10] Hempel de Ibarra N, Giurfa M, Vorobyev M (2002) Discrimination of coloured patterns by honeybees through chromatic and achromatic cues. J Comp Physiol A 188: 503–512. 10.1007/s00359-002-0322-x 12209339

[11] Neumeyer C (1981) Chromatic adaption in the honeybee: successive color contrast and color constancy. J Comp Physiol 144: 543–553.

[12] Dyer AG, Neumeyer C (2005) Simultaneous and successive colour discrimination in the honeybee (*Apis mellifera*). J Comp Physiol A 191: 547–557. 10.1007/s00359-005-0622-z 15871026

[13] Llandres AL, Rodriguez-Girones MA (2011) Spider Movement, UV Reflectance and Size, but Not Spider Crypsis, Affect the Response of Honeybees to Australian Crab Spiders. PLoS ONE 6: e17136. 21359183 10.1371/journal.pone.0017136 PMC3040225

[14] Heiling AM, Herberstein ME, Chittka L (2003) Pollinator attraction - Crab-spiders manipulate flower signals. Nature 421: 334–334. 12540891 10.1038/421334a

[15] Chittka L, Thomson JD, Waser NM (1999) Flower constancy, insect psychology, and plant evolution. Naturwiss 86: 361–377.

[16] Peitsch D, Fietz A, Hertel H, de Souza J, Ventura DF, et al (1992) The spectral input systems of hymenopteran insects and their receptor-based colour vision. J Comp Physiol A 170: 23–40. 1573568 10.1007/BF00190398

[17] Kulikowski JJ, Walsh V (1991) On the limits of colour detection and discrimination. In: Kulikowski JJ, Walsh V, Murray JJ, Cronly-Dillion JR, editors. Vision and visual dysfunction: Limits of vision. London: Macmillian. pp. 202–220.

[18] Helversen Ov (1972) Zur spektralen Unterschiedsempfindlichkeit der honigbiene. J Comp Physiol 80: 439–472.

[19] Brandt R, Vorobyev M (1997) Metric analysis of treshold spectral sensitivity in the honeybee. Vision Res 37: 425–439. 9156174 10.1016/s0042-6989(96)00195-2

[20] Dyer AG, Paulk AC, Reser DH (2011) Colour processing in complex environments: insights from the visual system of bees. Proc Royal Soc B 278: 952–959. 10.1098/rspb.2010.2412 PMC3049058 21147796

[21] Giurfa M (2004) Conditioning procedure and color discrimination in the honeybee *Apis mellifera* . Naturwiss 91: 228–231. 15146270 10.1007/s00114-004-0530-z

[22] Avarguès-Weber A, de Brito Sanchez MG, Giurfa M, Dyer AG (2010) Aversive reinforcement improves visual discrimination learning in free flying honeybees. PLOS One e15370. 20976170 10.1371/journal.pone.0015370 PMC2955543

[23] Dyer AG, Murphy AH (2009) Honeybees choose “incorrect” colors that are similar to target flowers in preference to novel colors. Isr J Plant Sci 57: 203–210.

[24] Giurfa M, Vorobyev M, Kevan P, Menzel R (1996) Detection of coloured stimuli by honeybees: minimum visual angles and receptor specific contrasts. J Comp Physiol A 178: 699–709.

[25] Giurfa M, Vorobyev M, Brandt R, Posner B, Menzel R (1997) Discrimination of coloured stimuli by honeybees: alternative use of achromatic and chromatic signals. J Comp Physiol A 180: 235–243.

[26] Lehrer M (1993) Parallel processing of motion, shape and colour in the visual system of the bee. In: Wiese K, editor. Sensory Systems of Arthropods. Basel: Birkhäuser Verlag.

[27] Ribi WA, Scheel M (1981) The second and third optic ganglia of the worker bee. Golgi studies of the neuronal elements in the medulla and lobula. Cell Tissue Res 221: 17–43. 7032703 10.1007/BF00216567

[28] Paulk AC, Dacks AM, Gronenberg W (2009) Color Processing in the Medulla of the Bumblebee (Apidae: *Bombus impatiens*). The Journal of Comparative Neurology 513: 441–456. 19226517 10.1002/cne.21993 PMC6783282

[29] Menzel R (1974) Spectral sensitivity of monopolar cells in the bee lamina. J Comp Physiol 93: 337–346.

[30] Ribi WA (1975) The first optic ganglion of the bee. I. Correlation between visual cell types and their terminals in the lamina and medulla. Cell Tiss Res 165: 103–111. 10.1007/BF00222803 1203968

[31] Ribi WA (1975) The neurons in the first optic ganglion of the bee (Apis mellifera). Advan Anat Embryol and Cell Biol 50: 1–43. 1199826

[32] Dyer AG, Spaethe J, Prack S (2008) Comparative psychophysics of bumblebee and honeybee colour discrimination and object detection. J Comp Physiol A 194: 617–627. 10.1007/s00359-008-0335-1 18437390

[33] Giurfa M, Lehrer M (2001) Honeybee vision and floral displays: from detection to close-up recognition. In: Chittka L, Thomson JD, editors. Cognitive Ecology of Pollination. Cambridge: University Press. pp. 61–82.

[34] Kaiser W, Liske E (1974) Die optomotorischen Reaktionen von fixiert fliegenden Bienen bei Reizung mit Spektrallichtern. J Comp Physiol 89: 391–408.

[35] Srinivasan MV, Lehrer M (1984) Temporal acuity of honeybee vision: behavioural studies using moving stimuli. J Comp Physiol A 155: 297–312.

[36] Stojcev M, Radtke N, D'Amaro D, Dyer AG, Neumeyer C (2011) General principles in motion vision: color-blindness of object motion depends on pattern velocity in honeybee and goldfish. Visual Neuroscience 28: 361–370. 21518470 10.1017/S0952523811000101

[37] Stach S, Bernard J, Giurfa M (2004) Local-feature assembling in the visual pattern recognition and generalization in honeybees. Nature 429: 758–761. 15201910 10.1038/nature02594

[38] Mota T, Yamagata N, Giurfa M, Gronenberg W, Sandoz JC (2011) Neural organization and visual processing in the anterior optic tubercle of the honeybee brain. J Neurosci 31: 11443–11456. 21832175 10.1523/JNEUROSCI.0995-11.2011 PMC6623125

[39] von Frisch K (1967) The Dance Language and Orientation of Bees. Cambridge: Harvard Univ. Press.

[40] Dyer AG (2007) Windy conditions affected colour discrimination in bumblebees (Hymenopteran: Apidea: Bombus). Entomol Gen 30: 165–166.

[41] Dyer AG (1998) The colour of flowers in spectrally variable illumination and insect pollinator vision. J Comp Physiol A 183: 203–212.

[42] Wyszecki G, Stiles WS (1982) Color science: Concepts and methods, quantitative data and formulae. New York: Wiley.

[43] Hempel de Ibarra N, Giurfa M, Vorobyev M (2001) Detection of coloured patterns by honeybees through chromatic and achromatic cues. J Comp Physiol A 187: 215–224. 11401201 10.1007/s003590100192

[44] Dyer AG, Chittka L (2004) Biological significance of distinguishing between similar colours in spectrally variable illumination: bumblebees (*Bombus terrestris*) as a case study. J Comp Physiol A 190: 105–114. 10.1007/s00359-003-0475-2 14652688

[45] Morawetz L, Spaethe J (2012) Visual attention in a complex search task differs between honeybees and bumblebees. J Exp Biol 215: 2515–2523. 22723491 10.1242/jeb.066399

[46] Dyer AG (1999) Broad spectral sensitivities in the honeybee's photoreceptors limit colour constancy. J Comp Physiol A 185: 445–453.

[47] Backhaus W (1991) Color opponent coding in the visual system of the honeybee. Vision Res 31: 1381–1397. 1891826 10.1016/0042-6989(91)90059-e

[48] Backhaus W, Menzel R, Kreissl S (1987) Multidimensional scaling of color similarity in bees. Biol Cybern 56: 293–304.

[49] Chittka L, Beier W, Hertel H, Steinmann E, Menzel R (1992) Opponent colour coding is a universal strategy to evaluate the photoreceptor inputs in hymentoptera. J Comp Physiol A 170: 545–563. 1507155 10.1007/BF00199332

[50] Menzel R (1967) Untersuchungen zum Erlernen von Spektralfarben durch die Honigbiene (*Apis mellifica*). Z Vergl Physiol 56: 22–62.

[51] Lehrer M, Bischof S (1995) Detection of model flowers by honeybees: the role of chromatic and achromatic contrast. Naturwiss 82: 145–147.

[52] Hempel de Ibarra N, Giurfa M (2003) Discrimination of closed coloured shapes by honeybees requires only contrast to the long wavelength receptor type. Anim Behav 66: 903–910.

[53] Niggebrugge C, Hempel de Ibarra N (2003) Colour-dependent target detection by bees. J Comp Physiol A 189: 915–918. 10.1007/s00359-003-0466-3 14614571

[54] Kelber A (2005) Alternative use of chromatic and achromatic cues in a hawkmoth. Proc R Soc B 272: 2143–2147. 10.1098/rspb.2005.3207 PMC1559944 16191627

[55] Lehrer M (1999) Dorsoventral asymmetry of colour discrimination in bees. J Comp Physiol A 184: 195–206.

[56] Neumeyer C (1980) Simultaneous color contrast in the honey bee. J Comp Physiol 139: 165–176.

[57] Vorobyev M, Brandt R (1997) How do insect pollinators discriminate colors? Isr J Plant Sci 45: 103–113.

[58] Hempel de Ibarra N, Vorobyev M, Brandt R, Giurfa M (2000) Detection of bright and dim colours by honeybees. J Exp Biol 203: 3289–3298. 11023849 10.1242/jeb.203.21.3289

[59] Dyer AG, Chittka L (2004) Fine colour discrimination requires differential conditioning in bumblebees. Naturwiss 91: 224–227. 15146269 10.1007/s00114-004-0508-x

[60] Dyer AG, Chittka L (2004) Bumblebees (*Bombus terrestris*) sacrifice foraging speed to solve difficult colour discrimination tasks. J Comp Physiol A 190: 759–763. 10.1007/s00359-004-0547-y 15316731

[61] Stach S, Giurfa M (2005) The influence of training length on generalization of visual feature assemblies in honeybees. Behav Brain Res 161: 8–17. 15904705 10.1016/j.bbr.2005.02.008

[62] Whitney HM, Kolle M, Andrew L, Chittka L, Glover BJ (2009) Floral iridescence, produced by diffractive optics, acts as a cue for animal pollinators. Science 323: 130–133. 19119235 10.1126/science.1166256

[63] Avarguès-Weber A, Portelli G, Benard J, Dyer A, Giurfa M (2010) Configural processing enables discrimination and categorization of face-like stimuli in honeybees. J Exp Biol 213: 593–601. 20118310 10.1242/jeb.039263

[64] Menzel R, Erber J, Masuhr T (1974) Learning and memory in the honeybee. In: Barton-Browne L, editor. Experimental analysis of insect behaviour. Berlin: Springer. pp. 195–217.

[65] Burns JG, Dyer AG (2008) Diversity of speed accuracy strategies benefits social insects. Curr Biol 18: R953–R954. 18957249 10.1016/j.cub.2008.08.028

[66] Kelber A (2010) What a hawkmoth remembers after hibernation depends on innate preferences and conditioning situation. Behav Ecol 21: 1093–1097.

[67] Camlitepe Y, Aksoy V (2010) First evidence of fine colour discrimination ability in ants (Hymenoptera, Formicidae). J Exp Biol 213: 72–77. 20008364 10.1242/jeb.037853

[68] Kelber A, Pfaff M (1999) True colour vision in the orchard butterfly, *Papilio aegeus* . Naturwiss 86: 221–224.

[69] Kunze J, Gumbert A (2001) The combined effect of color and odor on flower choice behavior of bumble bees in flower mimicry systems. Behav Ecol 12: 447–456.

[70] Jacobs GH, Williams GA, Cahill H (2007) Emergence of Novel Color Vision in Mice Engineered to Express a Human Cone Pigment. Science 315: 1723–1725. 17379811 10.1126/science.1138838

[71] Mancuso K, Hauswirth WW, Li Q, Connor TB, Kuchenbecker JA, et al (2009) Gene therapy for red–green colour blindness in adult primates. Nature 461: 784–787. 19759534 10.1038/nature08401 PMC2782927

[72] Paulk AC, Dacks AM, Phillips-Portillo J, Fellous JM, Gronenberg W (2009) Visual Processing in the Central Bee Brain. J Neurosci 29: 9987–9999. 19675233 10.1523/JNEUROSCI.1325-09.2009 PMC2746979

[73] Paulk AC, Phillips-Portillo J, Dacks AM, Fellous J, Gronenberg W (2008) The processing of colour, motion, and stimulus timing are anatomically segregated in the bumblebee brain. The Journal of Neuroscience 28: 6319–6332. 18562602 10.1523/JNEUROSCI.1196-08.2008 PMC3844780

[74] Paulk AC, Gronenberg W (2008) Higher order visual input to the mushroom bodies in the bee, Bombus impatiens. Arthropod Structure and Development 37: 443–458. 18635397 10.1016/j.asd.2008.03.002 PMC2571118

